# Supplementary material for: Ratio of the interferon-γ signature to the immunosuppression signature predicts anti-PD-1 therapy response in melanoma
Source: NPJ Genom Med. 2021 Feb 4;6:7. doi: 10.1038/s41525-021-00169-w (PMC7862369; doi:10.1038/s41525-021-00169-w)
Supplement: Supplementary file 1 — Supplementary Information [file 41525_2021_169_MOESM1_ESM.pdf]

Supplementary material for "Ratio of the interferon-γ signature to the immunosuppression signature predicts anti-PD-1 therapy response in melanoma"

C Cui et al.

Supplementary Fig. 1 | Association of IMS with abundance of immune cell types in TME on TCGA melanoma dataset ($n = 309$).

Supplementary Fig. 2 | Correlation analysis between IMS and IFN-γ, IMS and TMB on selected TCGA datasets. A-H, Pearson correlation analysis between IFN-γ signature and IMS scores, and between IFN-γ/IMS ratio and TMB, on the BRCA, COAD, LUAD, and SKCM datasets from TCGA.

Supplementary Fig. 3 | Log hazard ratio of IFN-γ/IMS score on selected TCGA datasets. Results are shown with 95% confidence intervals, with adjustment for sex (Female vs. Male), age (≥ 60 vs. < 60), tumor stage (III/IV vs. I/II) and TMB with a binary cutoff (top 20% of each cancer type). Cancers in which the IFN-γ/IMS ratio was statistically significantly ($p < 0.05$) associated with good prognosis are highlighted in blue; significant associations with poor prognosis are in red.

Supplementary Fig. 4 | Comparison of ORR from IFN-γ/IMS-high and IFN-γ/IMS-low patients on individual cohorts. a-f, Top: Comparison of ORR for patients from IFN-γ/IMS-high group versus patients from IFN-γ/IMS-low group with cutoff point (left), and for patients from IFN-γ-high group versus patients from IFN-γ-low group (right). Cutoff points were decided according to the Youden index on each cohort. Bottom: IFN-γ signature and IMS scores of individual patients in each cohort. Red and blue dashed lines indicate cutoff points for IFN-γ/IMS and IFN-γ signature score, respectively.

Supplementary Fig. 5 | t-SNE plot of cells from melanoma. Cells are colored by normalized expression of different IMS genes.

Supplementary Fig. 6 | Clinical responses to anti-PD-1 therapy The IFN-γ/IMS ratio scores of individual patients and their clinical responses to anti-PD-1 therapy.

Supplementary Table. 1: Genes in the immunosuppression signature

IMS biology	Gene	Putative function
Markers of immune cell	*FAP*, *PDGFRB*	CAFs
	CD163	Tumor-associated macrophages
	SIGLEC1 (*CD169*)	Tissue resident macrophages
Cytokines	*IL10*	Major immunosuppressive cytokine
	CCL2, *CCL8*, *CCL13*	chemokines that recruit immunosuppressive cells
Stromal factors	*INHBA*	Contribute to immune escape of tumours by inducing CAFs
	VCAN	Recruit and activate immunosuppressive myeloid cells; associated with a decrease in tumour-infiltrating cytotoxic T cells
	AXL	Promote EMT, tumour angiogenesis and inhibit antitumor immune response
	TWIST2, *ADAM12*	Promote tumour invasion and metastasis by inducing EMT and invadopodia-mediated ECM degradation
	COL6A3	Increase proliferation and decrease apoptosis in cancer cells; promote angiogenesis and inflammation
	STC1	Secreted by CAFs to enhance cancer cell intravasation and formation of distant metastases
	ISG15	Regulate immune response upon stimulation by type I IFNs; may act on cancer cells to reinforce their invasive capacity and tumorigenic potential
	BCAT1	Catalyse leucine transamination; may play a role in promoting cancer cell proliferation, migration and invasion
	OLFML2B	Potential oncogene related to regulation of the cell cycle, apoptosis and cell communication

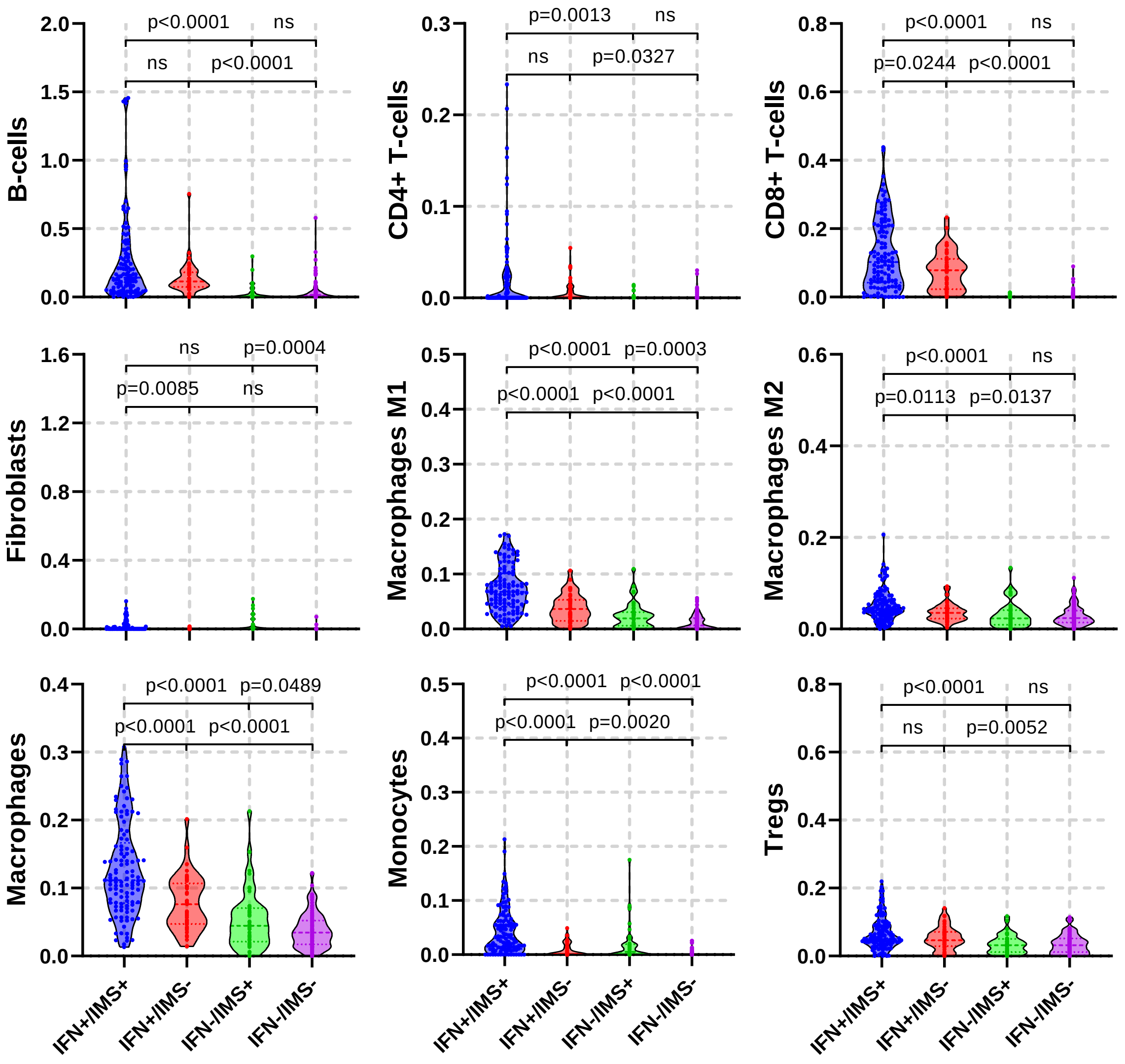

Supplementary Fig. 1:

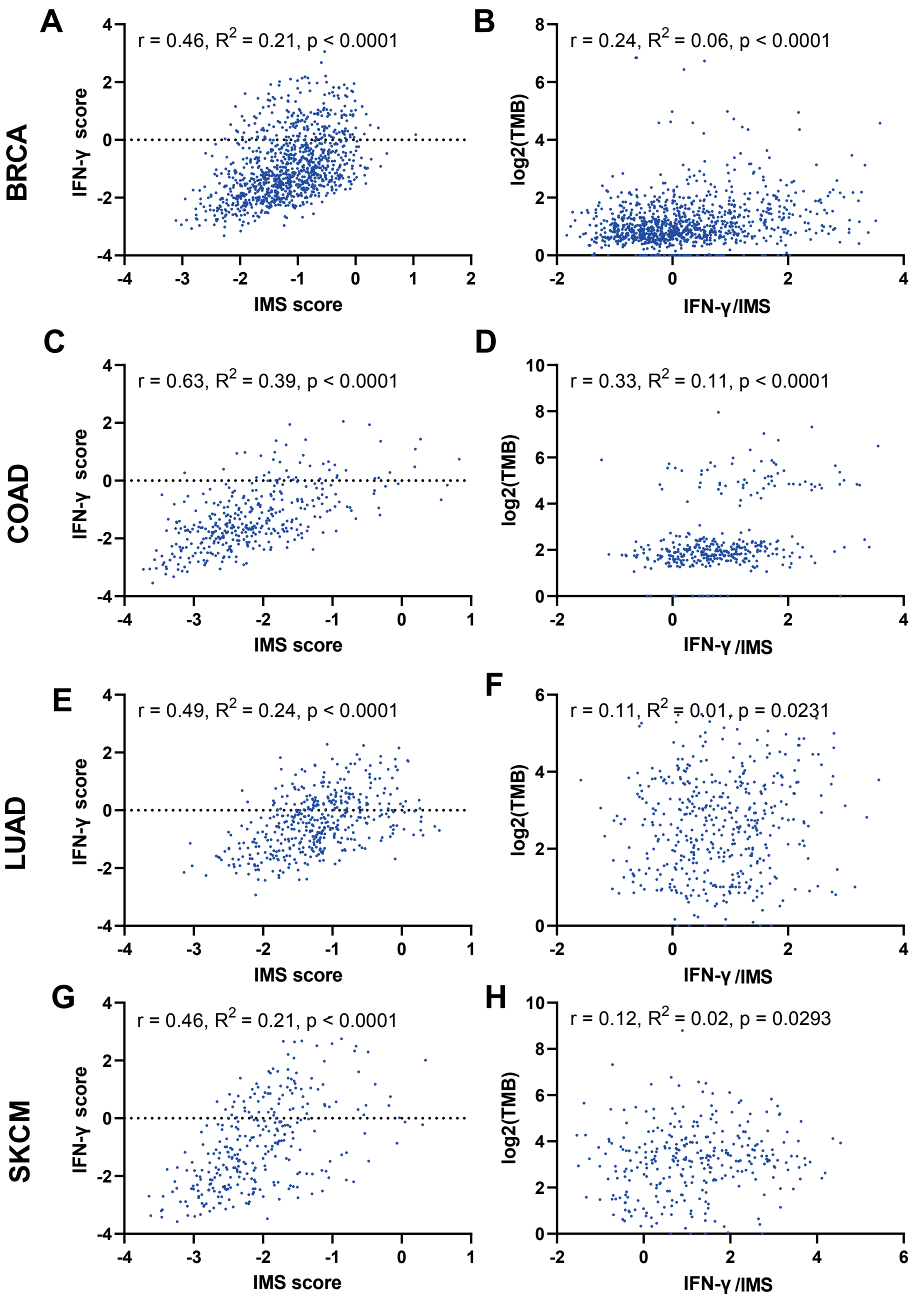

Supplementary Fig. 2:

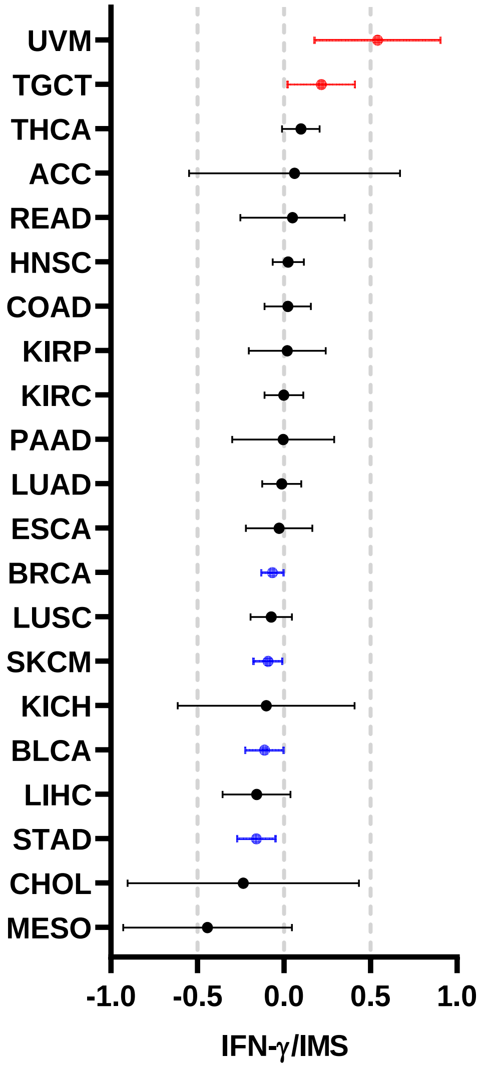

Supplementary Fig. 3:

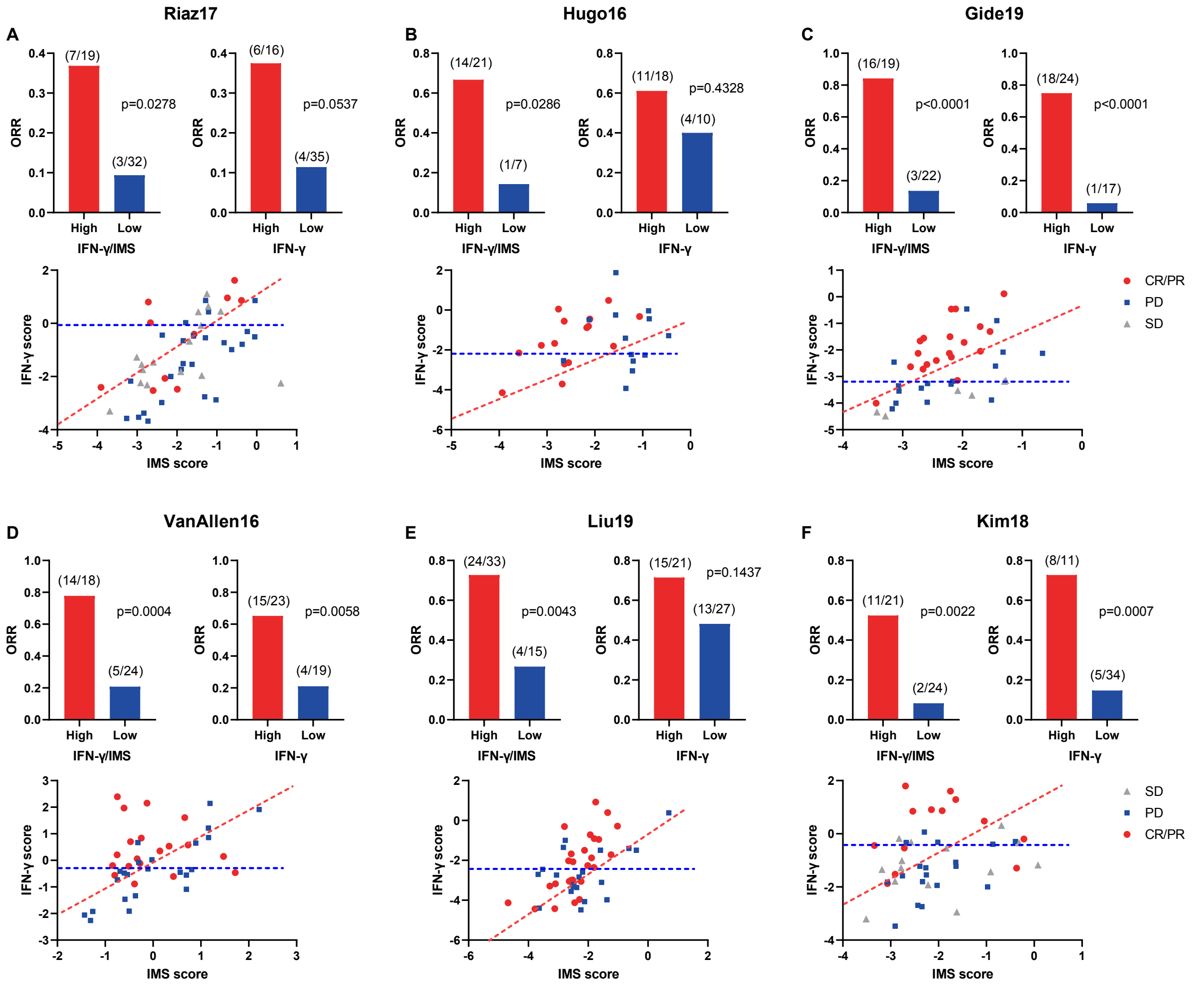

Supplementary Fig. 4:

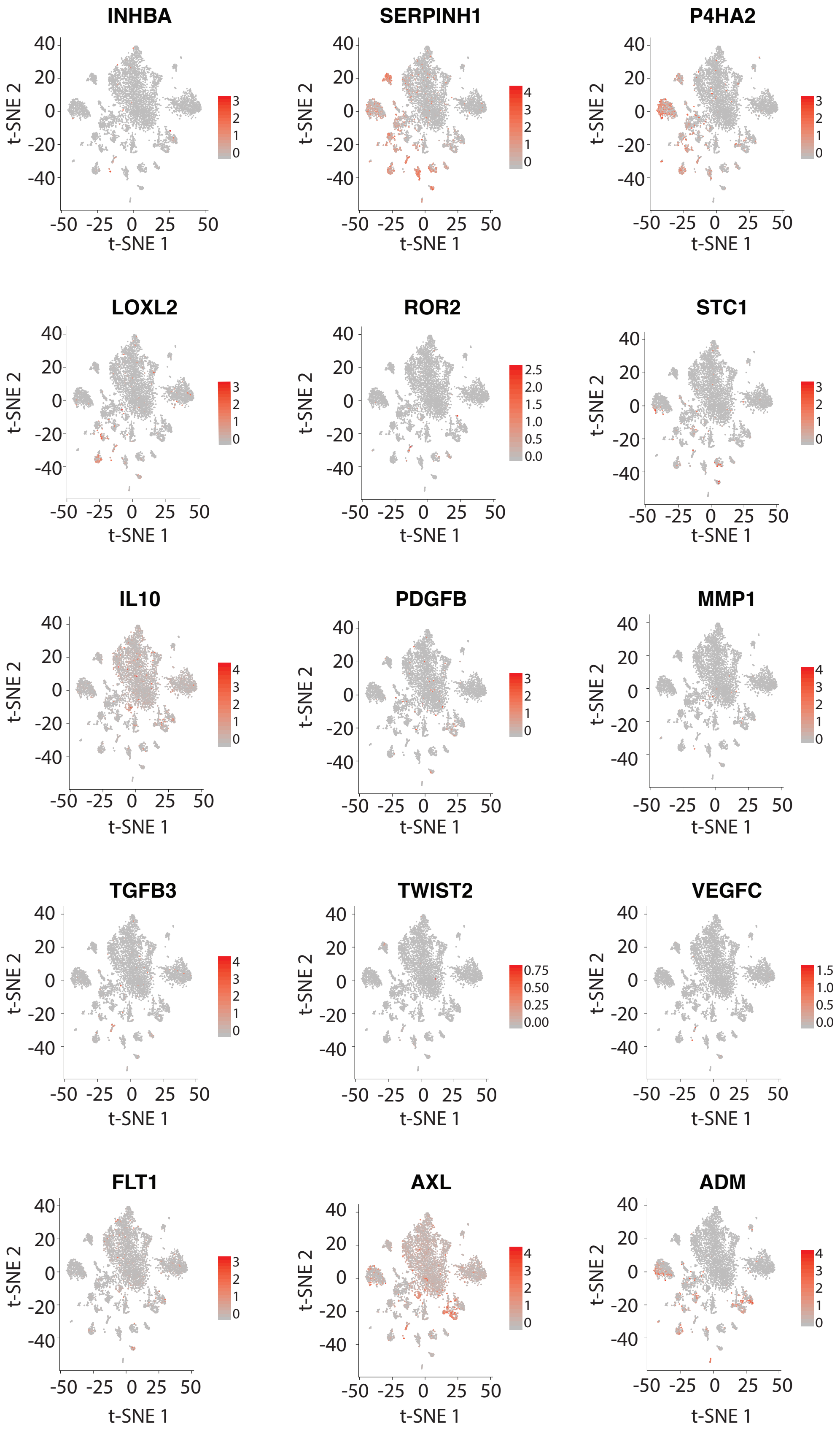

Supplementary Fig. 5:

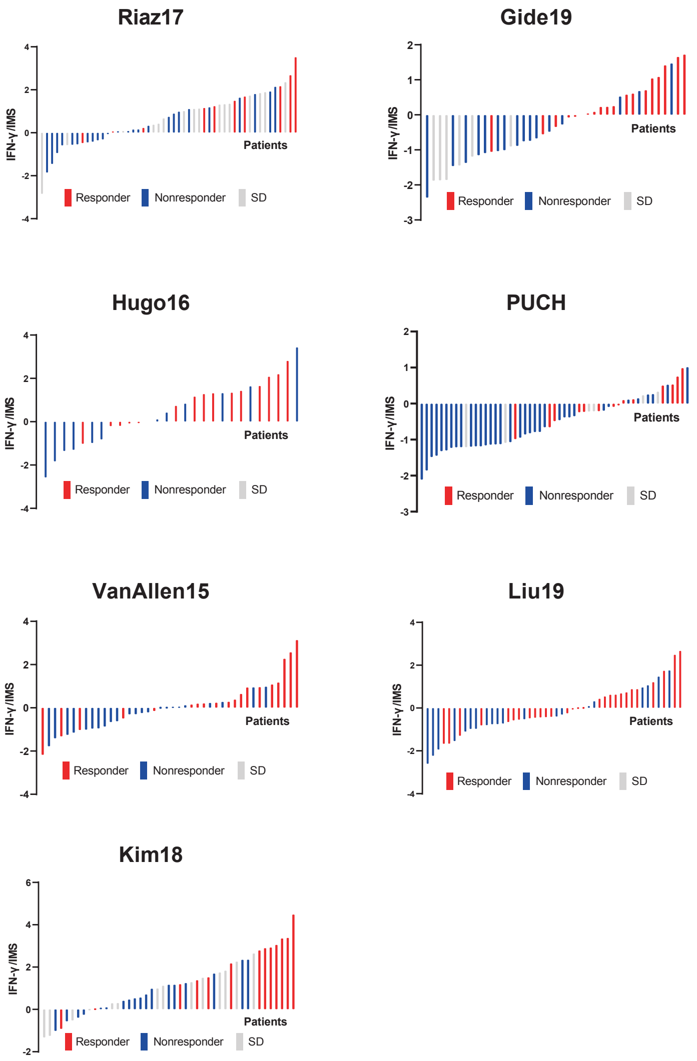

Supplementary Fig. 6:
