# Supplementary material for: Ratio of the interferon-γ signature to the immunosuppression signature predicts anti-PD-1 therapy response in melanoma
Source: NPJ Genom Med. 2021 Feb 4;6:7. doi: 10.1038/s41525-021-00169-w (PMC7862369; doi:10.1038/s41525-021-00169-w)
Supplement: Supplementary file 2 — Reporting Summary [file 41525_2021_169_MOESM2_ESM.pdf]

naturereserach

Corresponding author(s): Rongshan Yu
Last updated by author(s): Aug 15, 2020

Reporting Summary

Nature Research wishes to improve the reproducibility of the work that we publish. This form provides structure for consistency and transparency in reporting. For further information on Nature Research policies, see Authors & Referees and the Editorial Policy Checklist.

Statistics

For all statistical analyses, confirm that the following items are present in the figure legend, table legend, main text, or Methods section.

n/a	Confirmed	
☐	☒	The exact sample size (*n*) for each experimental group/condition, given as a discrete number and unit of measurement
☐	☒	A statement on whether measurements were taken from distinct samples or whether the same sample was measured repeatedly
☐	☒	The statistical test(s) used AND whether they are one- or two-sided<br>*Only common tests should be described solely by name; describe more complex techniques in the Methods section.*
☐	☒	A description of all covariates tested
☐	☒	A description of any assumptions or corrections, such as tests of normality and adjustment for multiple comparisons
☐	☒	A full description of the statistical parameters including central tendency (e.g. means) or other basic estimates (e.g. regression coefficient) AND variation (e.g. standard deviation) or associated estimates of uncertainty (e.g. confidence intervals)
☐	☒	For null hypothesis testing, the test statistic (e.g. *F*, *t*, *r*) with confidence intervals, effect sizes, degrees of freedom and *P* value noted<br>*Give P values as exact values whenever suitable.*
☒	☐	For Bayesian analysis, information on the choice of priors and Markov chain Monte Carlo settings
☒	☐	For hierarchical and complex designs, identification of the appropriate level for tests and full reporting of outcomes
☐	☒	Estimates of effect sizes (e.g. Cohen's *d*, Pearson's *r*), indicating how they were calculated

Our web collection on statistics for biologists contains articles on many of the points above.

Software and code

Policy information about availability of computer code

Data collection	The gene expression data of patients from PUCH cohort will be deposited in the NCBI database once the manuscript is accepted for publishing. All the other data supporting the findings of this study are available within the article and its Supplementary Information files. Raw sequencing data will be made available from the corresponding author upon reasonable request.
Data analysis	All analytical techniques are described and cited in the Methods section. Results generated using publicly available tools with parameters are described in Methods. Code to regenerate figures will be available in Github.<br>Software used:<br>Prism Graphpad (8.3.1)<br>Python (3.7.3)<br>STAR (2.6.1b)<br>RSEM (1.2.28)<br>xCell (1.1.0 under R 3.5.1)

For manuscripts utilizing custom algorithms or software that are central to the research but not yet described in published literature, software must be made available to editors/reviewers. We strongly encourage code deposition in a community repository (e.g. GitHub). See the Nature Research guidelines for submitting code & software for further information.

Data

Policy information about availability of data

All manuscripts must include a data availability statement. This statement should provide the following information, where applicable:

- Accession codes, unique identifiers, or web links for publicly available datasets
- A list of figures that have associated raw data
- A description of any restrictions on data availability

All patients data analysed from published papers are referenced to and publicly available accordingly. The gene expression data of patients from the PUCH cohort

will be deposited in the NCBI database once the manuscript is accepted for publishing. All the other data supporting the findings of this study are available within the article and its Supplementary Information files. Raw sequencing data will be made available from the corresponding author upon reasonable request.

Field-specific reporting

Please select the one below that is the best fit for your research. If you are not sure, read the appropriate sections before making your selection.

☒ Life sciences ☐ Behavioural & social sciences ☐ Ecological, evolutionary & environmental sciences

For a reference copy of the document with all sections, see nature.com/documents/nr-reporting-summary-flat.pdf

Life sciences study design

All studies must disclose on these points even when the disclosure is negative.

Sample size	No formal sample size calculation was performed for this retrospective cohort study. All available patients meeting clinical inclusion criteria who provided proper consents with available tumor tissue for sequencing were included in this study.
Data exclusions	No data from the PUCH cohort were excluded in our analysis. For external data sources, only data from patients who received anti-PD-1 monotherapy (Riaz17, Hugo16, Gide19, Kim18, Gide19, Liu19) or anti-CTLA-4 monotherapy (VanAllen15) and with necessary clinical information were used.
Replication	We performed replication of findings in three independent public datasets (Liu19, Kim18 and VanAllen15).
Randomization	Our study is a retrospective cohort study without randomization.
Blinding	Our study is a retrospective cohort study without blinding.

Reporting for specific materials, systems and methods

We require information from authors about some types of materials, experimental systems and methods used in many studies. Here, indicate whether each material, system or method listed is relevant to your study. If you are not sure if a list item applies to your research, read the appropriate section before selecting a response.

Materials & experimental systems

n/a	Involved in the study	
☒	☐	Antibodies
☒	☐	Eukaryotic cell lines
☒	☐	Palaeontology
☒	☐	Animals and other organisms
☐	☒	Human research participants
☐	☒	Clinical data

Methods

n/a	Involved in the study	
☒	☐	ChIP-seq
☒	☐	Flow cytometry
☒	☐	MRI-based neuroimaging

Human research participants

Policy information about studies involving human research participants

Population characteristics	Patients with age ranged from 27-73 suffer from advanced melanoma and received anti-PD-1 monotherapy. 32.7% were Male and 67.3% were Female. 18.2% had stage IIIc, 29.1% had M1a, 32.7% had M1b and 20.0% had M1c.
Recruitment	Patients were identified based on inclusion criteria (advanced melanoma, treated with anti-PD-1 monotherapy, tumor tissue available).
Ethics oversight	The study was approved by the Ethics committee of Peking University Cancer Hospital and Institute, Beijing, China.

Note that full information on the approval of the study protocol must also be provided in the manuscript.

Clinical data

Policy information about clinical studies

All manuscripts should comply with the ICMJE guidelines for publication of clinical research and a completed CONSORT checklist must be included with all submissions.

Clinical trial registration	Our study is a retrospective cohort study and no registration has been done.

Study protocol	Patients were required to suffer from advanced melanoma and to have received anti-PD-1 treatment, and have tissue obtained prior to anti-PD-1 treatment.
Data collection	Samples were collected at PUCH from 2016.3 - 2019.3.
Outcomes	Overall survival (OS) was defined as the time between first application of anti-PD-1 treatment and date of death at any cause. For subjects without documentation of death, OS was censored on the last date the subject was known to be alive. Progression-free survival (PFS) was defined as the time between first application of anti-PD-1 treatment and date of documented disease progression. For subjects without documentation of progression, PFS was censored on the last date the subject was known to be without progression. Response to anti-PD-1 treatment was assessed according to RECIST 1.1.
